# APC-targeted DNA vaccines: the role of CCL19 in immune cell recruitment and early onset of the immune response

**DOI:** 10.1007/s00262-026-04339-6

**Published:** 2026-03-10

**Authors:** Marina Barrio-Calvo, Stine Friis, Søren Vester Kofoed, Sofie Cens Holste, Rasmus Ohrt Andersen, Birgitte Rønø, Gertrud Malene Hjortø

**Affiliations:** 1 Department of Biomedical Sciences, University of Copenhagen, Copenhagen, Denmark; 2 Evaxion A/S, Hørsholm, Denmark

**Keywords:** APC-targeted DNA vaccines, CCL19, Cancer immunotherapy, Neoantigens

## Abstract

**Background:**

The introduction of DNA-encoded immune modulatory components is a promising strategy to enhance the immunogenicity of DNA vaccines. Antigen-presenting cell (APC)-targeted vaccines fuse DNA-encoded antigens with such adjuvants, fostering targeted immune activation. This study examined the cellular and molecular mechanisms of an APC-targeted DNA vaccine encoding Chemokine (C-C motif) ligand 19 (CCL19) fused to cancer neoantigens.

**Methods:**

DNA vaccines encoding CCL19 fused to a dimerization domain and cancer neoantigens were tested both in vitro and in vivo. CCR7-mediated G*α*i signaling, *β*-arrestin recruitment, and chemotaxis were evaluated in transfected cells and primary monocyte-derived dendritic cells. Protein expression and distribution were examined in vaccinated mice. The effect of CCL19 on vaccine-induced T-cell responses and anti-tumor efficacy was assessed in the CT26 syngeneic tumor model.

**Results:**

CCL19 retained its key biological functions when fused to cancer neoantigens, including CCR7-dependent signaling and chemotaxis of dendritic cells. In vivo, CCL19-fusion constructs were expressed locally and recruited immune cells to the immunization site. Tumor studies confirmed the superior immunogenicity and tumor control of the APC-targeted DNA vaccine, with CCL19 initiating an earlier immune response and enhancing anti-tumor effectiveness.

**Conclusions:**

CCL19 serves as an effective APC-targeting unit when fused to neoantigens, maintaining chemotactic and signaling properties that improve DNA vaccine immunogenicity and tumor control. This chemokine-mediated strategy offers a flexible approach to increase DNA vaccine potency with broad potential applications in cancer immunotherapies and beyond.

**Supplementary Information:**

The online version contains supplementary material available at 10.1007/s00262-026-04339-6.

## Background

Therapeutic cancer vaccines are immunotherapies designed to stimulate or enhance immune responses against tumor antigens. One promising modality for delivering tumor-specific antigens is the use of DNA vaccines, which consist of DNA plasmids encoding cancer antigens [1, 2].

Personalized cancer vaccines (PCVs) encode a selection of individualized tumor-specific antigens, called neoantigens, tailored to each patient’s tumor mutational landscape and chosen based on the patient’s unique HLA haplotype [3]. Several phase I and I/II clinical trials continue to demonstrate the ability of PCVs to elicit antigen-specific T-cell responses [4, 5]. Furthermore, the potential of PCVs to complement the clinical benefits of standard treatment therapies, such as checkpoint inhibitors, has been demonstrated in the phase II trial Keynote-942 [6].

In parallel with the selection of tumor antigens, efforts in DNA vaccine development focus on enhancing the overall immunogenicity of these vaccines. As a result, in addition to the neoantigens, a proportion of the DNA-based PCVs investigated in clinical trials incorporate DNA-encoded immune modulatory components (also referred to as molecular adjuvants) to modulate the induced immune responses. Molecular adjuvants, which include cytokines, co-stimulatory molecules of antigen-presenting cells (APC), or ligands of pathogen-recognition receptors, modulate the immune response through various mechanisms, activating innate immune responses or improving antigen presentation [7, 8].

Molecular adjuvants can be co-delivered in a separate plasmid, as exemplified in the phase II clinical trial NCT04251117, where patients receive two DNA plasmids encoding cancer antigens and interleukin-12 (IL-12) [4]. Alternatively, the molecular adjuvants can be fused to the cancer antigens in the same DNA construct, as seen in the clinical trials NCT05018273 and NCT01209871, where the cancer antigens are fused to chemokine (C-C motif) ligand 3 (CCL3) and CCL20, respectively [9]. When molecular adjuvants are covalently linked to antigens and capable of binding to APC receptors, facilitating antigen-APC interactions, the compound is categorized as an APC-targeted DNA vaccine [10].

Only a few professional APCs, such as dendritic cells (DCs) or macrophages, are transfected with the delivered DNA at the immunization site [11]. Therefore, APC-targeted DNA vaccines often include a secretion signal to release antigens into the extracellular milieu actively [12, 13], making them available for interaction with professional APCs through receptor-specific interactions, promoting superior immunogenicity [14].

Chemokines are especially promising as APC-targeting units. Beyond binding receptors in professional APCs, they influence the chemotaxis, differentiation, or maturation of the targeted cells [10, 15]. However, the complexity and promiscuity of the chemokine network pose challenges in unraveling their precise contribution to the efficacy of the APC-targeted vaccines.

The two natural ligands of Chemokine Receptor 7 (CCR7), CCL19 and CCL21, have demonstrated notable potential as APC-targeting units in preclinical models, with CCL19 exhibiting superior ability to induce antigen-specific T-cell responses and prevent tumor growth when fused to tumor antigens [16, 17]. CCL19 binding to CCR7, a G-protein-coupled receptor, initiates the G*α*i signaling pathway, regulating chemotaxis and cell survival, as well as *β*-arrestin recruitment, which mediates ligand-receptor internalization [18]. These mechanisms collectively govern DC migration and activation, controlling their ability to prime T cells.

We hypothesize that the enhanced immunogenicity of APC-targeted vaccines, particularly those containing CCL19, stems from properties intrinsic to the APC-targeting unit. In this study, we demonstrate that the signaling and chemotactic properties of CCL19 are preserved when it is included as an APC-targeting unit fused to cancer neoantigens, both in vitro and in vivo. Furthermore, we confirm the superiority of the APC-targeted vaccine “CCL19_CH3_Neo13”, a preclinical DNA vaccine encoding 13 cancer neoantigens (C1 to C13) fused to CCL19 [16], in inducing immunogenicity and tumor prevention when compared to a construct encoding only the cancer neoantigens, “Neo13” [19]. Our findings suggest that fusing cancer neoantigens to CCL19 promotes the interaction between them and professional APCs, leading to the superior efficacy observed in the murine model.

## Methods

### DNA designs

The DNA constructs used in this study are based on m/hCCL19_CH3_Neo13 [16]. m/hCCL19_CH3_Neo13 includes the pTVG4 DNA backbone plasmid encoding a fusion protein comprising i) murine or human (m/h) CCL19 as APC-targeting unit, ii) h1h4Ch3 (CH3) from human IgG3 as a dimerization domain, and iii) 13 27-mer CT26 in silico predicted neoepitopes (C1 to C13), hereon called Neo13, which have been extensively characterized in previous studies [19]. The “non-targeted” but secreted version of this plasmid, SS_CH3_Neo13, lacks the APC-binding molecule but includes the secretion signal (SS) derived from human CCL19 upstream of h1h4Ch3. The m/hCCL19_CH3 design encodes m/hCCL19 and h1h4Ch3 but lacks the antigenic cassette, and m/hCCL19_CH3_Neo2 includes only the first two cancer neoantigens (C1 and C2). Finally, the Neo13 DNA construct encodes the same neoantigens cloned into the pTVG4 backbone plasmid, without additional elements [19]. The empty backbone plasmid pTVG4 is referred to as mock DNA throughout this study. The DNA constructs were upscaled to in vivo-grade DNA by either Aldevron, Cobra, or GeneArt.

### Recombinant fusion proteins

The fusion protein encoded by the hCCL19_CH3 DNA construct was purchased from GenScript, which recombinantly expressed it at a concentration of 1.2 µg/ml using the CHO-K1 cell line as the expression system.

### Animal experiments

Animal experiments were conducted under license 2022-15-0201-01318 from the Danish Animal Experiments Inspectorate, in accordance with the Danish Animal Experimentation Act (BEK nr. 12 of 7/01/2016), which is compliant with the European Directive (2010/63/EU). Experiments were conducted at Evaxion A/S´s animal facility on 8–14-week-old wild-type BALB/c JrJ female mice purchased from Janvier Labs (France). Each cage was considered a randomization unit and contained a balanced representation of all treatment groups. All procedures were performed one cage at a time by technical personnel blinded to group allocation. Mice were euthanized by cervical dislocation.

### DNA immunization

#### Copolymer formulation

DNA was formulated with the co-block polymer poloxamer 188 [19]. For studies of immune cell infiltration into the vaccination site, mice were immunized intramuscularly (i.m.) in the right and left tibialis anterior muscles with a single dose of 90 µg of DNA. For tumor development studies, mice received a total of four or five immunizations of 1–6 µg of DNA at weekly intervals in the same anatomical location following a prophylactic schedule relative to tumor cell inoculation (defined as study day zero). Mice were immunized at study days ( − 14), − 7, 0, 7, and 14.

#### Electroporation (EP)-assisted vaccination

In experiments designed to investigate the biodistribution of the DNA-encoded fusion proteins, mice received one i.m. injection of 50 µg of DNA formulated in PBS without calcium or magnesium in the gastrocnemius muscle. Immediately after intramuscular DNA injection, mice were electroporated using an AgilePulse system (BTX, Harvard Apparatus) with a two-phase pulse protocol: a single high-voltage conditioning pulse (450 V, 0.05 ms) followed by eight low-voltage delivery pulses (110 V, 10 ms) separated by 20 ms intervals. Device logs confirmed accurate pulse delivery and impedance reduction between cycles, consistent with effective poration.

### Tumor experiments

BALB/c mice were injected subcutaneously (s.c.) in the right flank with 2 × 10^5^ CT26 cells formulated 1:1 in Matrigel (Corning, #354,234). The tumor volume was evaluated manually by blinded technical personnel three times per week and calculated as tumor volume = $\frac{\pi }{6}{(\mathrm{d}1*\mathrm{d}2)}^{3/2}$ where d1 and d2 represent tumor diameters. When depicting tumor volumes over time, missing data were corrected by applying Last-Observation-Carried-Forward (LOCF) [19].

### Organ collection and processing

#### Spleen collection and processing into single-cell suspensions for fluorescence-activated cell sorting (FACS) analysis

For analysis of immunogenicity of DNA vaccines, mice were euthanized, and spleens from a representative number of mice were collected in cold RPMI (Gibco, #72,400-021) supplemented with 10% heat-inactivated fetal bovine serum (FBS) (from here on: R10 media) and processed into single-cell suspensions using GentleMACS supplies (Miltenyi Biotec, C-tubes, #130,096,334 and Dissociator, #130,093,235), and cryopreserved until use in FBS supplemented with 10% DMSO. Upon thawing in pre-warmed R10 media, the viability of a representative number of samples (15% of the thawed splenocytes) was evaluated using a Nucleocunter NC-200 (Chemometec). Samples display an average viability of approximately 75%.

#### Muscle collection and processing into single-cell suspensions for FACS analysis

For analysis of immune infiltration, immunized mice were euthanized 2, 4, 6, or 8 days after DNA immunization, and muscles at the injection site were collected as described by Oprescu et al. [20]. Their weight was normalized prior to processing into single-cell suspensions via enzymatic dissociation (Miltenyi, #130,098,305), and stained with fluorochrome-labeled antibodies, including FITC anti-CD45.2 (BD, #561,874), PerCP-Cy5.5 anti-CD3 (BioLedgend, #100,328), PE-Cy7 anti-CD19 (BD, #561,739), BV711 anti-CD11b (BD, #563,168), BV421 anti-CD11c (BD, #565,451), BV605 anti-MHC-II (BioLedgend, #107,639) prior to analysis by flow cytometry [21].

#### Muscle and serum collection and processing for investigating the biodistribution of DNA-encoded fusion proteins

Muscles were homogenized in T-Per buffer (ThermoFisher, #78,510) using GentleMACS supplies (Miltenyi, M-tubes, #130,093,236 and Dissociator, #130,093,235) for protein extraction. Whole blood was collected from the retroorbital plexus into silica-coated tubes, incubated for one hour at room temperature, and centrifuged at 2000 g for 10 min before serum collection. Protein extracts from muscle lysates and serum were cryopreserved for later use.

### Peptide re-stimulation and intracellular cytokine staining (ICS)

Neoantigen-reactive CD8 + and CD4 + T cells were detected in spleens re-stimulated with synthetic neo-peptides (5 µg/ml) corresponding to the vaccine antigens, followed by ICS and flow cytometry analysis. Synthetic peptides (27-mer) featuring the mutated amino acid in the central position were produced by GenScript (New Jersey, USA).

Frequencies of interferon γ (IFN γ) and tumor necrosis factor *α* (TNF* α*)-producing CD4 + and CD8 + T cells were analyzed in samples stained with fluorescently labeled anti-CD3, anti-CD8, and anti-CD4 antibodies and a viability dye by flow cytometry as previously described [19]. Samples with fewer than 500 CD8 + T cells were excluded from further analysis. The gating strategy is found in the supplementary material (SFig. 2).

### MHC I multimer staining for detection of neoantigen-specific T cells

To analyze neoantigen-specific CD8 + T cells, 50 µl of sublingual blood was collected in heparin-coated tubes and stained with a fluorochrome-labeled MHC class I tetramer specific for the neoantigen C1 (H-2K^d^ loaded with KFKASRASI, C1 minimal epitope) together with fluorescently-labeled lineage antibodies, including anti-CD3, anti-CD8, and anti-CD4 antibodies, before being analyzed by flow cytometry as previously described [19]. The gating strategy is found in the supplementary material (SFig. 3).

### Cell lines

CT26 cells (ATCC, #CRL2638) were cultured in R10 media. HEK293 and HEK293T cells were cultured in DMEM (Merck, #D6546) supplemented with 10% FBS and 1% Penicillin/Streptomycin. All cell lines were maintained at 37 °C and 5% CO2.

### DNA transfection and collection of conditioned media

One million HEK293T cells seeded in 6-well plates were transfected with one µg of DNA constructs using six µl of Lipofectamine 2000 (11,668,027, ThermoFisher) in Opti-Mem media (Gibco, 31,985,062) [16]. After five hours, the media was exchanged to Opti-Mem. Supernatants, hereon called conditioned media, were collected 24 h later.

### Sandwich ELISA and immunoblotting

The expression of APC-targeted DNA plasmids was assessed by human CCL19 DuoSet sandwich ELISA (R&D Systems, #DY361) in conditioned media, serum, and protein extracts from muscle lysates, as per the manufacturer’s instructions. In muscle lysates, the total protein concentration was previously normalized by BCA analysis (Thermofisher, #23,225).

The molecular weights of the proteins present in the muscle lysates were verified by immunoblotting. The samples reduced with β-mercaptoethanol (Sigma-Aldrich, #63,689) were run on 20% Mini-Protean TM TGX Stain-Free Protein Gels (Bio-Rad, #4,568,093), transferred to PVDF membranes, and incubated with primary and secondary antibodies targeting hCCL19 (R&D Systems, #BAU0821021 and #HAF017) [16]. Immunoblotting of conditioned media utilized a Mouse anti-Human IgG (CH3 domain) Secondary Antibody (Invitrogen, #MA5-16,557) as the primary antibody and Anti-mouse IgG HRP-conjugated (R&D Systems, #HAF007) as the secondary antibody, as previously described [16].

### Bioluminescence-resonance energy transfer (BRET) measurement of G*α*i signaling and *β*-arrestin recruitment.

BRET (bioluminescence resonance energy transfer) assays were used to evaluate G*α*i signaling and *β*-arrestin recruitment [22]. 500.000 HEK293 cells/well were transfected with constructs encoding human CCR7 and the CAMYEL sensor (cAMP sensor using Rluc-Epac-YFP) for G*α*i signaling, or Rluc8-Arr3 fusion and MEM-citrine-SH3 for *β*-arrestin recruitment using Lipofectamine 2000 (11,668,027, ThermoFisher). Transfected cells were collected in PBS with high glucose 24 h after and treated with coelenterazine (5 µM) (355, Nanolight) for 10 min, followed by the addition of varying concentrations of recombinant hCCL19_CH3 protein or recombinant hCCL19, which were incubated for 5 min. For BRET measurements of G*α*i signaling, forskolin (F6886, Sigma) was added at this point. Thirty-five minutes after the addition of the ligands, BRET signals were measured as YFP (530 nm)/Rluc (480 nm) bioluminescence using a PerkinElmer Envision reader.

### Generation of monocyte-derived DCs and chemotaxis

Monocytes were isolated from human peripheral blood mononuclear cells (PBMCs) by plastic adhesion and differentiated into immature moDCs using interleukin 4 (IL-4) (250 U/mL, 200-04, Peprotech) and granulocyte–macrophage colony-stimulating factor (GM-CSF) (1000 U/mL, 300-03, Peprotech) for six days. The cells were further matured into moDCs using interleukin 6 (IL-6) (1000 U/mL, 200-06, Peprotech), interleukin 1*β* (IL-1*β*) (1000 U/mL, 200-01B, Peprotech), TNF-*α* (1000 U/mL, 300-01A, Peprotech), and prostaglandin 2 (PGE2) (1 μg/mL, P0409, Sigma) for an additional 2 days [23]. MoDCs were frozen in 90% human serum and 10% DMSO until used.

- Chemotaxis in trans-well chambers: MoDCs were incubated with an anti-CCR7 antibody or isotype control antibody (20 µg/ml) for 15 min before loading into the insert (40.000 cells/insert) of 96-trans-well chambers (5 µm pore size) (Corning #3388) where they were allowed to migrate toward conditioned media containing APC-targeted proteins for 60 min at 37 °C. Antibodies were also added to the conditioned media. Transmigrated cells were quantified using the CellTiter Glo kit (Promega #G7570).
- Three-dimensional (3D) chemotaxis: MoDCs (3 × 10^6^ cells/ml) were embedded in collagen I (1.67 mg/ml) and loaded into µ-Slide Chemotaxis chambers (Ibidi #80,326). After gelation, the sink reservoirs were filled with media from untransfected cells and the source reservoirs were filled with conditioned media containing APC-targeted proteins. Cell migration was monitored using video microscopy over 16 h at 2-min intervals in a time-lapse microscope equipped with a humidified, temperature-controlled stage incubator. Cell migration was tracked using a commercial tracking program (Autozell) and subsequently analyzed to get a population-based chemotactic index (CI) value in MATLAB [22]. Individual cell movement was visualized using Python in spider diagrams.

### Statistical analysis

GraphPad Prism 9 for macOS was used to generate the graphs and perform the statistical analyses. The data were subjected to the Shapiro–Wilk test for normality (*α* = 0.05). Parametric data were analyzed using One-Way ANOVA with Tukey’s correction for multiple comparisons. Non-parametric data were analyzed by the Kruskal–Wallis test with Dunn’s multiple comparison correction. For the tests described above, the following levels of statistical significance were applied: ns *p* ≥ 0.05, *p < 0.05, **p < 0.01, ***p < 0.001, ****p < 0.0001.

## Results

### Signaling and chemotactic properties of CCL19-fusion proteins through CCR7

**Fig. 1:**
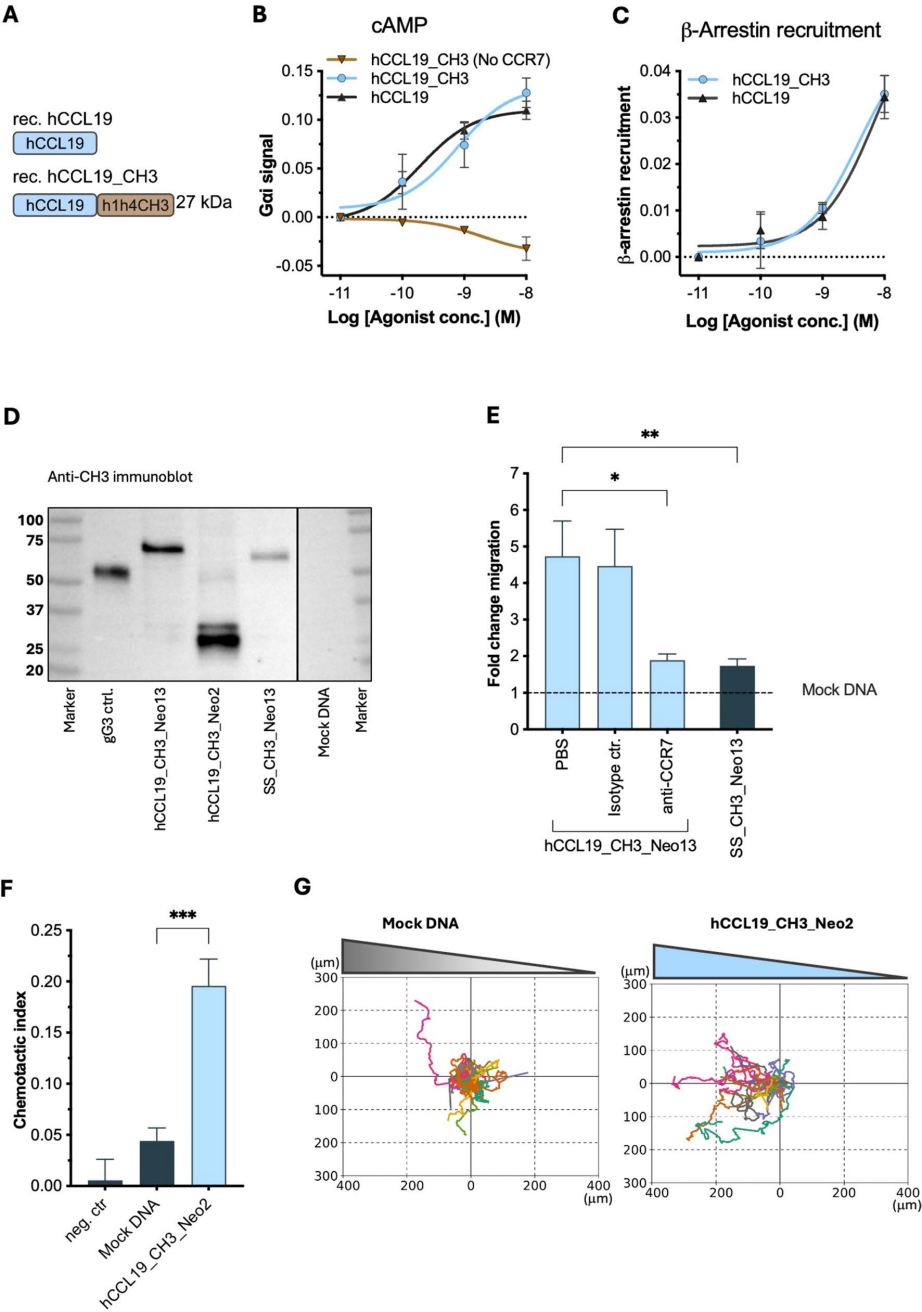
Signaling and chemotactic properties of CCL19 fusion proteins. **A** Schematic representation of the recombinant proteins used for these experiments. **B–C** Signaling properties of recombinant hCCL19_CH3 fusion protein in CCR7-transfected HEK293 cells as (**B**) G⍺i signaling and (**C**) *β*-arrestin recruitment assessed using BRET-based assays. Dose–response curves were generated from a three-parameter logistic regression model. **D** Immunobloting analysis detecting the dimerization domain CH3 in conditioned media (reduced) of transfected HEK293T cells. **E** CCR7-dependent migration of moDCs toward conditioned media containing hCCL19_CH3_Neo13 in Boyden chambers, n = 6. **F** Chemotactic index (CI) quantifying the migration of moDC populations toward conditioned media containing hCCL19_CH3_Neo2 and with mock DNA over 16 h, assessed using time-lapse microscopy and **G** spider diagrams showing the trajectory of individual moDCs, n = 3. Statistics (**E–F**): One-way ANOVA with Tukey’s correction for multiple tests. Significance: *p < 0.05, **p < 0.01, ***p < 0.001

To elucidate the mechanism of action by which APC-targeted vaccines encoding CCL19 result in improved immunogenicity and anti-tumor efficacy, we examined whether the signaling properties of CCL19 remain functional when linked to the dimerization domain h1h4CH3 by testing the ability of CCL19-fusion proteins to engage with the receptor CCR7. We demonstrate that the hCCL19_CH3 protein (27 kDa) (Fig. 1A) induces CCR7-mediated G*α*i signaling and *β*-arrestin recruitment in a dose-dependent manner in HEK293 cells transiently transfected with CCR7, with a half-maximal effective concentration (EC50) comparable to hCCL19 (G*α*i signaling EC50 ~ 0.8 versus 0.2 nM and *β*-arrestin recruitment EC50 ~ 3.8 versus 7.3 nM) (Fig. 1B–C). hCCL19_CH3 was used for this assay because it was not possible to synthesize a high enough concentration of the antigen-encoding fusion protein mCCL19_CH3_Neo13.

To substantiate these findings, we examined the chemotactic ability of hCCL19-fusion proteins using monocyte-derived DCs (moDCs). This experiment evaluated the chemotactic capability of conditioned media generated from HEK293T transfected cells containing hCCL19_CH3_Neo2, hCCL19_CH3_Neo13, and the non-targeted counterpart SS_CH3_Neo13, whose concentration and their correct molecular weight were evaluated using a sandwich ELISA against hCCL19 and immunoblotting against CH3 (Fig. 1D).

Conditioned media containing hCCL19_CH3_Neo13 (0.1 nM) induced a two-fold increase in migration compared to SS_CH3_Neo13 in a Boyden chamber assay. Furthermore, the migration caused by hCCL19_CH3_Neo13 was successfully abrogated by an anti-CCR7 blocking antibody, indicating the specificity of the CCL19-CCR7 interaction in the induction of chemotaxis (Fig. 1E). Additionally, moDC showed robust and directed migration in response to the linear chemotactic gradient of mCCL19_CH3_Neo2 (2 nM) established in a three-dimensional gel matrix designed to preserve the physiological behavior and migratory capabilities of moDCs (Fig. 1F–G).

These findings suggest that CCL19’s signaling through CCR7 and the migration events resulting from that are maintained when incorporated into APC-targeted DNA vaccines. Furthermore, including hCCL19 as an APC-targeting unit could play a crucial role in favoring the encounter of DCs with the antigens.

### In vivo expression and localized presence of APC-targeted fusion proteins post-DNA vaccination

To gain insights into the distribution of hCCL19_CH3-derived fusion proteins after immunization with the DNA constructs, we evaluated the presence of the translated protein products in muscle lysates and serum seven and 14 days after one intramuscular (i.m.) EP-assisted immunization with 50 µg of DNA. To avoid detection of endogenous CCL19, mice were immunized with DNA vaccines encoding human CCL19, which shares 83% sequence identity with the murine counterpart and retains the ability to signal through murine CCR7 [24, 25].

**Fig. 2:**
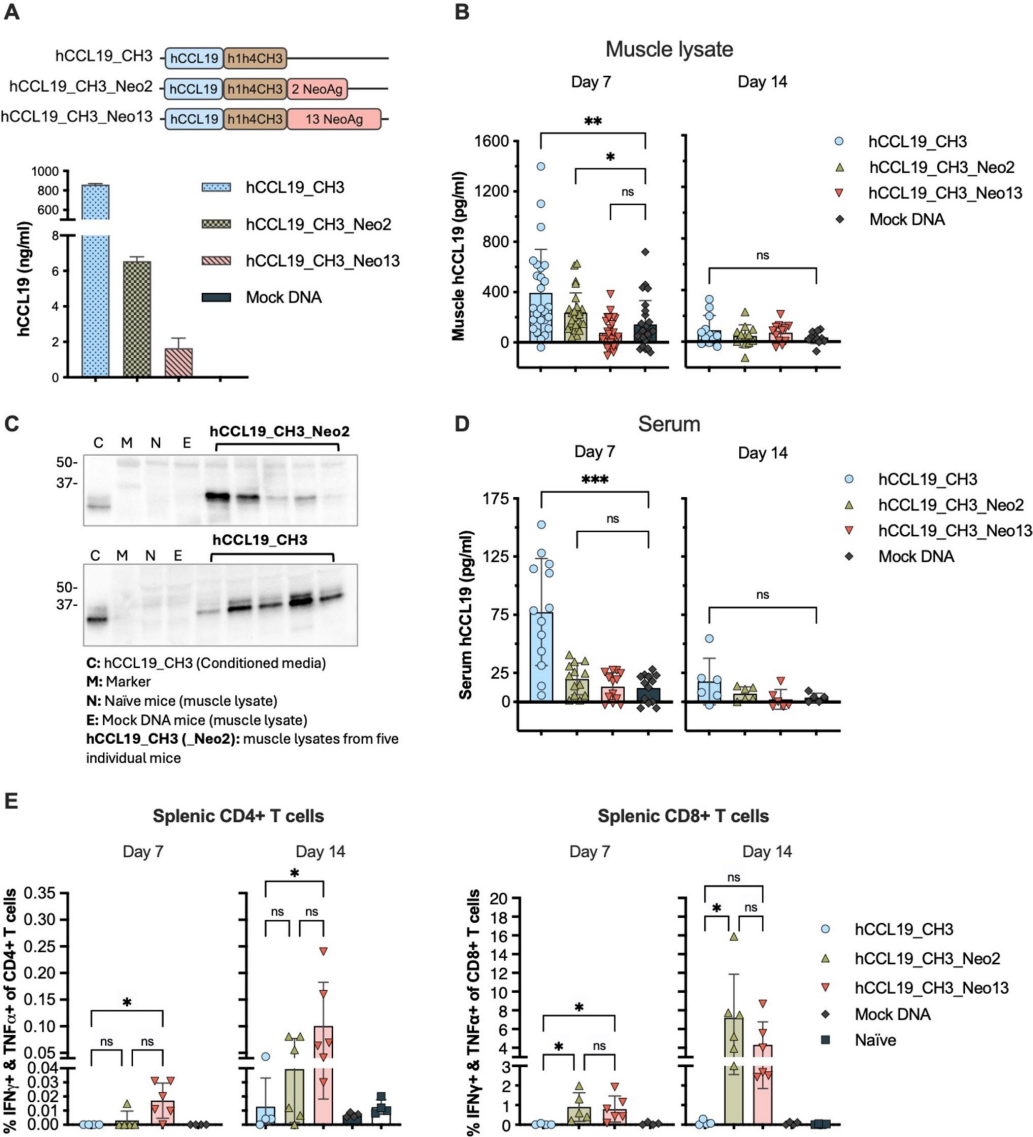
In vivo expression of CCL19 fusion proteins after DNA immunization. **A** Schematic representation of the DNA constructs encoding different variations of hCCL19_CH3_Neo13 and evaluation of their expression and secretion measured in the supernatant of transiently transfected HEK293T cells by hCCL19 sandwich ELISA. **B–C** hCCL19 fusion proteins measured in the muscle lysates of individual BALB/c mice 7 or 14 days after DNA immunization, assessed by (**B**) hCCL19 sandwich ELISA (data from two independent experiments amounting for a total of 56 mice. 5–7 animals/group, n (muscle lysate) = 28. Mean ± SD) or (**C**) by immunobloting against hCCL19 7 days after DNA immunization (5 muscle lysates per group). **D** hCCL19 fusion proteins in serum 7 or 14 days after DNA immunization were analyzed by hCCL19 sandwich ELISA (data from two independent experiments, 5–7 animals/group, n (mice) = 14. Mean ± SD). **E** IFN γ and TNF*α* double-secreting CD4 + (left) and CD8 + (right) T cells in the spleen of mice 7 or 14 days after DNA immunization were analyzed by ICS and flow cytometry. Statistics: Kruskal–Wallis test with Dunn’s multiple comparison correction. Significance: ns *p* > 0.05, *p < 0.05, **p < 0.01, ***p < 0.001

After seven days, both hCCL19_CH3 and hCCL19_CH3_Neo2 proteins were detected in muscle lysates in the range of 400 and 200 pg/ml, respectively via hCCL19 ELISA (Fig. 2B). These results were corroborated by immunoblotting against hCCL19, confirming the presence and correct molecular weight of both fusion proteins (Fig. 2C). As expected, hCCL19_CH3_Neo13 was not detected either by ELISA or immunoblotting (data not shown), as the addition of a longer neoantigen cassette negatively impacts protein expression [25]. In serum, the hCCL19_CH3 protein was present at 75 pg/ml (Fig. 2D), whereas the larger hCCL19_CH3_Neo2 protein could not be identified. At study day 14, hCCL19_CH3 and hCCL19_CH3_Neo2 were no longer detectable in muscle lysates or serum, indicating clearance of the protein products from these compartments.

Despite hCCL19_CH3_Neo13 being undetectable in both tissues, its functional activity was demonstrated by the induction of vaccine-specific CD4 + and CD8 + functional T cells. The frequencies of CD8 + reactive T cells were comparable among both treatment groups (Fig. 2E right), while hCCL19_CH3_Neo13 resulted in a higher frequency of reactive CD4 + T cells (Fig. 2E left) driven by the presence of additional strong MHC class II-restricted binders in the Neo13 antigen cassette compared to Neo2 [19].

### mCCL19_CH3-derived DNA constructs recruit immune cells to the vaccination site

We examined whether APC-targeted DNA vaccines can leverage the chemotactic properties of CCL19 in vivo to recruit immune cells to the immunization site after DNA vaccination.

**Fig. 3:**
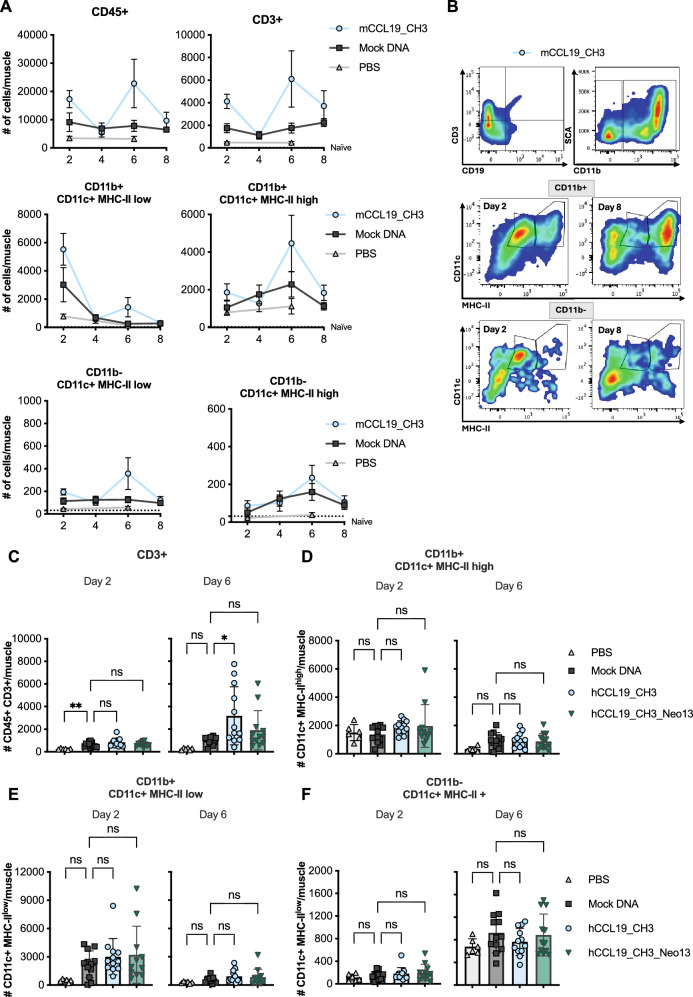
Recruitment of immune cells to the immunization site by mCCL19_CH3-derived DNA constructs **A** Quantification of immune cells per muscle at two, four, six, or eight days after one i.m. immunization with 90 μg of DNA formulated with poloxamer 188. The number of immune cells (CD45 +), T-cells (CD45 + CD3 + CD19-), and DCs (CD45 + CD3-CD19-CD11b + CD11c + MHC-II +) were quantified by flow cytometry (mean ± SD, 34 mice distributed as 2–3 mice/group, n (muscle lysates) = 4–6). **B** Gating strategy for A). **C–E** Quantification of immune cells per muscle two and six after one i.m. immunization with 90 μg of DNA in a subsequent experiment (mean ± SD, 42 mice distributed as 3–6 mice/group, n (muscle lysates) = 6–12). Statistics (**C–E**): One-way ANOVA with Tukey’s correction for multiple tests. Significance: ns *p* > 0.05, *p < 0.05, **p < 0.01

Six days after one i.m. immunization with 90 µg of the mCCL19_CH3 DNA, FACS analysis of skeletal muscle immune infiltrates revealed a trend toward increased immune cell (CD45 +) infiltration in mice receiving mCCL19_CH3 (Fig. 3A upper-left). Notably, T cells (CD45 + CD3 + CD19-) were significantly enriched compared to the mock DNA group (Fig. 3A upper-right). Similarly, DCs (CD45 + CD3-CD19-CD11c + MHC-II +) were more abundant in the mCCL19_CH3 group at this time point. As previously reported, within the myeloid population, we find a large fraction of cells that co-express the markers CC11b and CD11c [21], with CD11b- cells being less abundant. Within the DC compartment of mice immunized with mCCL19_CH3, pre-DCs (CD11b + CD11c + MHC-II low) peaked two days after immunization (mCCL19_CH3 mean: 5.530 cells/muscle vs mock DNA mean: 3.020 cells/muscle). By day six, DCs with a more mature phenotype characterized by higher expression of MHC-II had become the dominant subset (mCCL19_CH3 ~ 4.460 cells/muscle vs mock DNA ~ 2.280 cells/muscle) (Fig. 3A middle-right & B). This tendency was also observed in DC populations negative for CD11b, in which the number of cells expressing high levels of MHC-II increased at day 6 post-vaccination (Fig. 3A lower-right & B). Poloxamer 188 was selected for DNA formulation to minimize nonspecific inflammation and immune cell recruitment induced by electroporation. Nonetheless, more immune cells are present in Mock DNA-treated mice than in the naïve group, indicating a certain level of unspecific immune cell recruitment.

A subsequent experiment confirmed the recruitment of T cells to the immunization site six days post-immunization with CCL19-encoding APC-targeted DNA constructs (mCCL19_CH3 and mCCL19_CH3_Neo13) when compared to mock DNA (Fig. 3C left). While we observed a decrease in pre-DC populations from days two to six (Fig. 3D left), the differences previously observed between the APC-targeted constructs and the mock DNA could not be reproduced, and no significant increase in mature DCs was detected in this experiment (Fig. 3E).

While these findings suggest a role for CCL19 as an APC targeting unit in recruiting immune cells to the immunization site, there is an evident need for further optimization of the experimental setups, as well as a better understanding of the molecular cues orchestrating the migration of DCs to the skeletal muscle.

### CCL19 and the dimerization domain h1h4CH3 contribute to an early onset of the immune response, improving the tumor-protection capabilities of APC-targeted DNA vaccines

Previous studies have shown how APC-targeted DNA vaccines drive an earlier onset of the vaccine-specific immune response [16, 21], as well as revealing the role of the secretion signal in enhancing immunogenicity [16]. To further investigate the role of different DNA elements in the improved efficacy of APC-targeted DNA vaccines, we compared the efficacy of Neo13, a DNA cancer vaccine encoding the same 13 neoantigens, with SS_CH3_Neo13, which maintains CCL19’s secretion signal and incorporates the dimerization domain h1h4CH3. We also evaluated the impact of adding mCCL19 as an APC-targeting unit and as a molecular adjuvant (delivered via a separate plasmid) on the immunogenicity and tumor prevention ability of the cancer neoantigens.

Mice received five i.m. immunizations with 3 µg of DNA formulated with poloxamer 188 in weekly intervals. Two immunizations were performed before the CT26 tumor inoculation (study day zero). The induction of neoantigen-specific T cells was monitored on study day 4 (SFig. 1A).

The co-delivery of mCCL19 with Neo13 did not improve the immunogenicity of the encoded neoantigens, evidenced by comparable levels of C1-specific CD8 + T cells in circulation between Neo13 and mCCL19 + Neo13 groups (SFig. 1C). Similarly, both groups achieved partial tumor prevention (SFig. 1B). In comparison, SS_CH3_Neo13 elicited significantly higher neoantigen-specific T-cell responses (SFig. 1B and C). The addition of mCCL19 to this plasmid (mCCL19 + SS_CH3_Neo13) further increased the frequency of neoantigen-specific T-cells (1.3% C1-specific CD8 + T cells), to a level comparable with that induced by CCL19_CH3_Neo13. (SFig. 1C). These findings suggest that introducing both a secretion signal upstream of the cancer antigens and mCCL19 either as an APC-targeting unit or as a molecular adjuvant improves their ability to prime antigen-specific immune responses. However, complete tumor prevention of mCCL19_CH3_Neo13 and mCCL19 + SS_CH3_Neo13 at the tested DNA dose indicates that this experimental setup lacks sufficient resolution to precisely dissect the potential contributions of CCL19 as an APC-targeting unit to the anti-tumor efficacy.

**Fig. 4:**
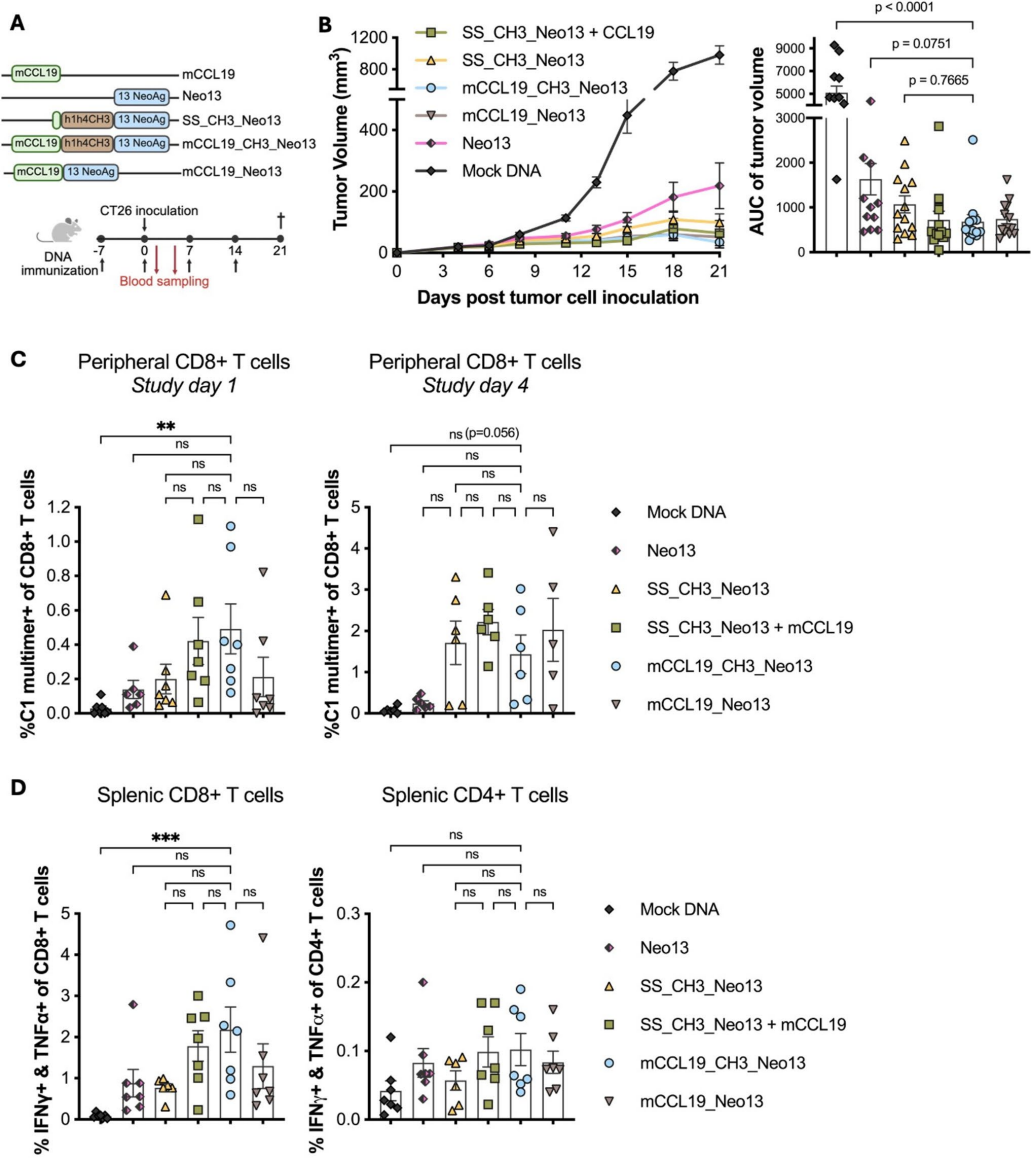
Immunogenicity and anti-tumor efficacy of cancer neoantigens improve upon adding CCL19 as an APC-targeting unit and h1h4CH3 as a dimerization domain. **A** The schematic design of the study outline and the DNA construct used in B-D. BALB/c mice (a total of 78 mice distributed as: n = 13 mice/group) received four weekly immunizations of 3 or 6 μg of DNA prophylactically. One week after the first immunization, mice were inoculated with 2 × 10^5^ CT26 tumor cells s.c. in the right flank (defined as study day 0). On study days 1 and 4, the frequency of C1-specific T cells was evaluated in peripheral blood. The study was terminated 21 days after tumor inoculation. **B** Mean tumor volume (mm3) ± SEM with last observation carried forward (LOCF) over time and mean AUC ± SD of individual tumors for each treatment group. **C** Frequency of C1-specific CD8 + T cells. Mean ± SD (n = 6–7 mice per group). (**D**) IFN γ and TNF*α* double-secreting CD4 + and CD8 + splenic T cells analyzed by flow cytometry after re-stimulation with vaccine containing epitopes and ICS (n = 4–7 mice per group). Statistics: Kruskal–Wallis test and Dunn ´s multiple comparison test. All comparisons performed are displayed. **p < 0.01, ***p < 0.001

Seeking to unveil these differences, we performed an additional experiment were only one (out of four) DNA immunizations was delivered before the CT26 tumor cell inoculation (Fig. 4A). Similarly to the setup described above, mice were treated with Neo13, SS_CH3_Neo13 delivered alone or in combination with mCCL19, mCCL19_CH3_Neo13, and a monomeric version of this last construct, mCCL19_Neo13 in an attempt to investigate further the impact of the dimerization domain h1h4CH3 in the immunogenicity and anti-tumor efficacy of the APC-targeted DNA vaccine (Fig. 4A).

The results showed that while SS_CH3_Neo13 and CCL19_Neo13 are remarkably potent, achieving tumor protection in most of the animals (Fig. 4B), these treatments fail to raise antigen-specific T cells above Neo13 at study day one (eight days after the first immunization). In contrast, ~ 0.5% of the CD8 + T cells were specific for C1 in mice treated with SS_CH3_Neo13 + mCCL19 and mCCL19_CH3_Neo13 (Fig. 4C left), indicating a potential synergy between mCCL19 and h1h4CH3 in inducing an earlier onset of antigen-specific T cells. Three days after, at study day four, animals receiving SS_CH3_Neo13, SS_CH3_Neo13 + mCCL19, mCCL19_CH3_Neo13 and mCCL19_Neo13 display a strong increase in C1-specific CD8 + T cells indicating that while the breadth of the immune response is similar in all groups, the onset of the immune response is highly defined by the different elements encoded in the DNA plasmids (Fig. 4C right). Upon termination of the study, SS_CH3_Neo13 + mCCL19 and mCCL19_CH3_Neo13 led to similar levels of reactive antigen-specific CD8 + T cells, which were mildly higher than in the Neo13, SS_CH3_Neo13, and mCCL19_Neo13 groups in alignment with the results obtained by the blood staining (Fig. 4D left).

## Discussion

In this study, we corroborated key aspects of the proposed mechanism of action by which CCL19, as an APC-targeting unit, improves the immunogenicity and anti-tumor efficacy of DNA-encoded cancer neoantigens. We demonstrate that in this context, CCL19 retains its functional properties, including CCR7-mediated signaling and chemotaxis of immune cells, such as T cells and DCs. Furthermore, the APC-targeted DNA vaccine, mCCL19_CH3_Neo13, containing murine CCL19 and a set of CT26 previously validated neoantigens, achieved total tumor control in a syngeneic tumor mouse model where the addition of CCL19 and h1h4Ch3 to the DNA design significantly shortened the onset of the immune response.

The hCCL19_CH3 protein activated CCR7-dependent G*α*i signaling and β-arrestin recruitment, essential pathways in chemokine signaling. Chemotaxis assays showed that hCCL19_CH3_Neo2 and hCCL19_CH3_Neo13 induced significant migration of primary human moDCs, mediated through CCR7-dependent mechanisms, thereby affirming the biological activity of the engineered chemokine construct. These results align with previous findings showing that C-terminal CCL19-fusion proteins retain functional properties, such as receptor engagement and downstream signaling [26]. Together, these data highlight that hCCL19_CH3_Neo13 can leverage the CCL19-CCR7 pathway to enhance DNA vaccine efficacy through multiple mechanisms, including the efficient secretion of antigens and the recruitment of immune cells.

The successful expression and secretion of CCL19-fusion proteins in vivo were confirmed by detecting these proteins in muscle lysates and serum following DNA vaccination. Additionally, the APC-targeted molecules improved the recruitment of immune cells to the immunization site, including DCs and T cells, although future research should focus on elucidating the molecular cues governing the migration of immune cells in response to DNA immunization, as this aspect remains understudied. Together, these findings suggest that the fusion proteins are expressed and secreted into the extracellular environment, where they can interact with immune cells, thereby facilitating antigen presentation and immune cell activation.

The relatively low immunogenicity of DNA vaccines has been a significant limitation in the development of these therapies. Utilizing chemokines as molecular adjuvants to increase the recruitment, expansion, and activation of APCs at the immunization site is an appealing approach to improve their tumor control capabilities in preclinical models [8, 16, 17, 27–29]. In line with this hypothesis, tumor studies conducted under a prophylactic vaccination schedule demonstrated that mCCL19_CH3_Neo13 significantly inhibited tumor growth in the CT26 syngeneic model and highlighted CCL19’s capacity to promote early onset of vaccine-induced immune responses. Given the aggressive nature of the CT26 tumor model, selecting a prophylactic schedule provided a controlled framework to delineate the contributions of the different elements of CCL19 biology when used as an APC-targeting unit, including the functional importance of the secretion signal. The data presented here suggest that the secretion signal plays a significant role in the overall vaccine efficacy. However, the current experimental setup limited our ability to discriminate more nuanced differences between constructs and prevented definitive conclusions. Therefore, future studies should focus on identifying a suboptimal DNA dose, testing alternative immunization schedules, including therapeutic vaccination schedules, and additional tumor models with growth kinetics conducive to assessing the individual contribution of the components of the APC-targeting unit.

This study’s findings are consistent with prior research demonstrating the utility of chemokines as molecular adjuvants to augment DNA vaccine-induced immunity. Notably, recent clinical studies have begun to incorporate cytokines as APC-targeting units, often in combination with complementary strategies such as checkpoint inhibitors or second-generation DNA delivery platforms.

In conclusion, our findings highlight the potential of CCL19-mediated APC-targeting DNA vaccines in improving the immunogenicity and anti-tumor efficacy of cancer vaccines. By leveraging the dual chemotactic and CCR7-signaling properties of CCL19, we have demonstrated that these vaccines can effectively recruit and activate immune cells, leading to improved tumor control of the cancer antigens. A significant advantage of this approach is its versatility. By altering the neoantigen cassette, the construct can be customized to target various tumor antigens. This modularity positions APC-targeted DNA vaccines as a promising platform for a wide range of immunological applications.

## Supplementary Information

Below is the link to the electronic supplementary material.

Supplementary file1 (DOCX 575 kb)

## Data Availability

The datasets generated during and/or analysed during the current study are available from the corresponding author on reasonable request.

## References

[1] Fan T, Zhang M, Yang J, Zhu Z, Cao W, Dong C (2023) Therapeutic cancer vaccines: advancements, challenges and prospects. Signal Transduct Target Ther 8:1–23. 10.1038/s41392-023-01674-3 38086815 10.1038/s41392-023-01674-3 PMC10716479

[2] Chi W-Y, Hu Y, Huang H-C, Kuo H-H, Lin S-H, Kuo C-TJ et al (2024) Molecular targets and strategies in the development of nucleic acid cancer vaccines: from shared to personalized antigens. J Biomed Sci 31:1–26. 10.1186/s12929-024-01082-x 39379923 10.1186/s12929-024-01082-x PMC11463125

[3] Liu J, Fu M, Wang M, Wan D, Wei Y, Wei X (2022) Cancer vaccines as promising immuno-therapeutics: platforms and current progress. J Hematol Oncol 15:1–26. 10.1186/s13045-022-01247-x 35303904 10.1186/s13045-022-01247-x PMC8931585

[4] Yarchoan M, Gane EJ, Marron TU, Perales-Linares R, Yan J, Cooch N et al (2024) Personalized neoantigen vaccine and pembrolizumab in advanced hepatocellular carcinoma: a phase 1/2 trial. Nat Med 30:1044–1053. 10.1038/s41591-024-02894-y 38584166 10.1038/s41591-024-02894-y PMC11031401

[5] Viborg N, Kleine-Kohlbrecher D, Rønø B (2024) Personalized Neoantigen DNA Cancer Vaccines: Current Status and Future Perspectives. J Cell Immunol 6:15–24. 10.33696/immunology.6.188

[6] Weber JS, Carlino MS, Khattak A, Meniawy T, Ansstas G, Taylor MH et al (2024) Individualised neoantigen therapy mRNA-4157 (V940) plus pembrolizumab versus pembrolizumab monotherapy in resected melanoma (KEYNOTE-942): a randomised, phase 2b study. Lancet 403:632–644. 10.1016/S0140-6736(23)02268-7 38246194 10.1016/S0140-6736(23)02268-7

[7] Sabbaghi A, Ghaemi A (2021) Molecular adjuvants for DNA vaccines DNA vaccines: application, design, preparation, and formulation. In: Sousa Â, editor. DNA Vaccines Methods Protoc [Internet]. New York, NY: Springer US; [cited 2023 July 27]. p. 87–112. 10.1007/978-1-0716-0872-2_5 10.1007/978-1-0716-0872-2_5 32827133

[8] Kutzler MA, Weiner DB (2004) Developing DNA vaccines that call to dendritic cells. J Clin Invest 114:1241. 10.1172/JCI23467 15520855 10.1172/JCI23467 PMC524242

[9] Szymura SJ, Wang L, Zhang T, Cha S, Song J, Dong Z et al (2024) Personalized neoantigen vaccines as early intervention in untreated patients with lymphoplasmacytic lymphoma: a non-randomized phase 1 trial. Nat Commun 15:6874. 10.1038/s41467-024-50880-2 39128904 10.1038/s41467-024-50880-2 PMC11317512

[10] Biragyn A, Tani K, Grimm MC, Weeks S, Kwak LW (1999) Genetic fusion of chemokines to a self tumor antigen induces protective, T-cell dependent antitumor immunity. Nat Biotechnol 17:253–258. 10.1038/6995 10096292 10.1038/6995

[11] Doe B, Selby M, Barnett S, Baenziger J, Walker CM (1996) Induction of cytotoxic T lymphocytes by intramuscular immunization with plasmid DNA is facilitated by bone marrow-derived cells. Proc Natl Acad Sci U S A 93:8578–8583. 10.1073/pnas.93.16.8578 8710913 10.1073/pnas.93.16.8578 PMC38715

[12] Hohlfeld R, Engel AG (1994) The immunobiology of muscle. Immunol Today 15:269–274. 10.1016/0167-5699(94)90006-X 8068173 10.1016/0167-5699(94)90006-X

[13] Ulmer JB, Deck RR, Dewitt CM, Donnelly JJ, Liu MA (1996) Generation of MHC class I‐restricted cytotoxic T lymphocytes by expression of a viral protein in muscle cells: antigen presentation by non‐muscle cells. Immunology 89:59–67. 10.1046/j.1365-2567.1996.d01-718.x 8911141 10.1046/j.1365-2567.1996.d01-718.x PMC1456656

[14] Løvås T-O, Bruusgaard JC, Øynebråten I, Gundersen K, Bogen B (2014) DNA Vaccines: MHC II-Targeted Vaccine Protein Produced by Transfected Muscle Fibres Induces a Local Inflammatory Cell Infiltrate in Mice. PLoS ONE. 10.1371/journal.pone.0108069 25299691 10.1371/journal.pone.0108069 PMC4191975

[15] Ruffini PA, Grødeland G, Fredriksen AB, Bogen B (2010) Human chemokine MIP1α increases efficiency of targeted DNA fusion vaccines. Vaccine 29:191–199. 10.1016/J.VACCINE.2010.10.057 21055498 10.1016/j.vaccine.2010.10.057

[16] Barrio-Calvo M, Kofoed SV, Holste SC, Sørensen AB, Viborg N, Kringelum JV, et al. (2023) Targeting neoantigens to APC-surface molecules improves the immunogenicity and anti-tumor efficacy of a DNA cancer vaccine. Front Immunol [Internet]. [cited 2023 Sept 7]; 14. https://www.frontiersin.org/articles/10.3389/fimmu.2023.1234912. Accessed 7 Sept 2023 10.3389/fimmu.2023.1234912 PMC10499626 37720215

[17] Qi H, Sun Z, Gao T, Yao Y, Wang Y, Li W et al (2024) Genetic fusion of CCL11 to antigens enhances antigenicity in nucleic acid vaccines and eradicates tumor mass through optimizing T-cell response. Mol Cancer 23:46. 10.1186/s12943-024-01958-4 38459592 10.1186/s12943-024-01958-4 PMC10921619

[18] Zhang M, Chen T, Lu X, Lan X, Chen Z, Lu S (2024) G protein-coupled receptors (GPCRs): advances in structures, mechanisms and drug discovery. Signal Transduct Target Ther 9:1–43. 10.1038/s41392-024-01803-6 38594257 10.1038/s41392-024-01803-6 PMC11004190

[19] Viborg N, Pavlidis MA, Barrio-Calvo M, Friis S, Trolle T, Sørensen AB et al (2023) DNA based neoepitope vaccination induces tumor control in syngeneic mouse models. Npj Vaccines 8:1–16. 10.1038/s41541-023-00671-5 37244905 10.1038/s41541-023-00671-5 PMC10224666

[20] Oprescu SN, Yue F, Kuang S (2020) Single-cell isolation from regenerating murine muscles for RNA-sequencing analysis. STAR Protoc 1:100051. 10.1016/j.xpro.2020.100051 33111097 10.1016/j.xpro.2020.100051 PMC7580090

[21] Langlet C, Tamoutounour S, Henri S, Luche H, Ardouin L, Grégoire C et al (2012) CD64 expression distinguishes monocyte-derived and conventional dendritic cells and reveals their distinct role during intramuscular immunization. J Immunol 188:1751–1760. 10.4049/jimmunol.1102744 22262658 10.4049/jimmunol.1102744

[22] Brandum EP, Jørgensen AS, Calvo MB, Spiess K, Peterson FC, Yang Z et al (2022) Selective boosting of CCR7-acting chemokines; short peptides boost chemokines with short basic tails, longer peptides boost chemokines with long basic tails. Int J Mol Sci 23:1397. 10.3390/ijms23031397 35163323 10.3390/ijms23031397 PMC8836243

[23] Hansen M, Met Ö, Larsen NB, Rosenkilde MM, Andersen MH, Svane IM et al (2016) Autocrine CCL19 blocks dendritic cell migration toward weak gradients of CCL21. Cytotherapy 18:1187–1196. 10.1016/J.JCYT.2016.06.010 27424146 10.1016/j.jcyt.2016.06.010

[24] Nguyen-Hoai T, Hohn O, Vu MD, Baldenhofer G, Sayed Ahmed MS, Dörken B et al (2012) CCL19 as an adjuvant for intradermal gene gun immunization in a Her2/neu mouse tumor model: improved vaccine efficacy and a role for B cells as APC. Cancer Gene Ther 19:880–887. 10.1038/cgt.2012.78 23099886 10.1038/cgt.2012.78

[25] Kim CH, Pelus LM, White JR, Applebaum E, Johanson K, Broxmeyer HE (1998) CK beta-11/macrophage inflammatory protein-3 beta/EBI1-ligand chemokine is an efficacious chemoattractant for T and B cells. J Immunol Baltim Md 1950 160:2418–2424 9498785

[26] Purvanov V, Matti C, Samson GPB, Kindinger I, Legler DF (2018) Fluorescently tagged CCL19 and CCL21 to monitor CCR7 and ACKR4 functions. Int J Mol Sci 19:3876. 10.3390/ijms19123876 30518137 10.3390/ijms19123876 PMC6321256

[27] Nguyen‐Hoai T, Baldenhofer G, Ahmed MS, Pham‐Duc M, Gries M, Lipp M et al (2012) CCL19 (ELC) improves TH1‐polarized immune responses and protective immunity in a murine Her2/neu DNA vaccination model. J Gene Med 14:128–137. 10.1002/jgm.1651 22228591 10.1002/jgm.1651

[28] Sumida SM, McKay PF, Truitt DM, Kishko MG, Arthur JC, Seaman MS et al (2004) Recruitment and expansion of dendritic cells in vivo potentiate the immunogenicity of plasmid DNA vaccines. J Clin Invest 114:1334–1342. 10.1172/JCI200422608 15520866 10.1172/JCI22608 PMC524232

[29] Liu J, Kjeken R, Mathiesen I, Barouch DH (2008) Recruitment of antigen-presenting cells to the site of inoculation and augmentation of human immunodeficiency virus type 1 DNA vaccine immunogenicity by in vivo electroporation. J Virol 82:5643–5649. 10.1128/JVI.02564-07 18353952 10.1128/JVI.02564-07 PMC2395223

