# Correction: APC-targeted DNA vaccines: the role of CCL19 in immune cell recruitment and early onset of the immune response

**DOI:** 10.1007/s00262-026-04412-0

**Published:** 2026-07-11

**Authors:** Marina Barrio-Calvo, Stine Friis, Søren Vester Kofoed, Sofie Cens Holste, Rasmus Ohrt Andersen, Birgitte Rønø, Gertrud Malene Hjortø

**Affiliations:** 1 Department of Biomedical Sciences, University of Copenhagen, Copenhagen, Denmark; 2 Evaxion A/S, Hørsholm, Denmark

**Correction to: Cancer Immunology, Immunotherapy (2026) 75:101** 10.1007/s00262-026-04339-6

In the original version of this article, panel letters A, B, C etc were missing in the Fig. 3 and the Fig. 3 should have appeared as shown below.

**Fig. 3:**
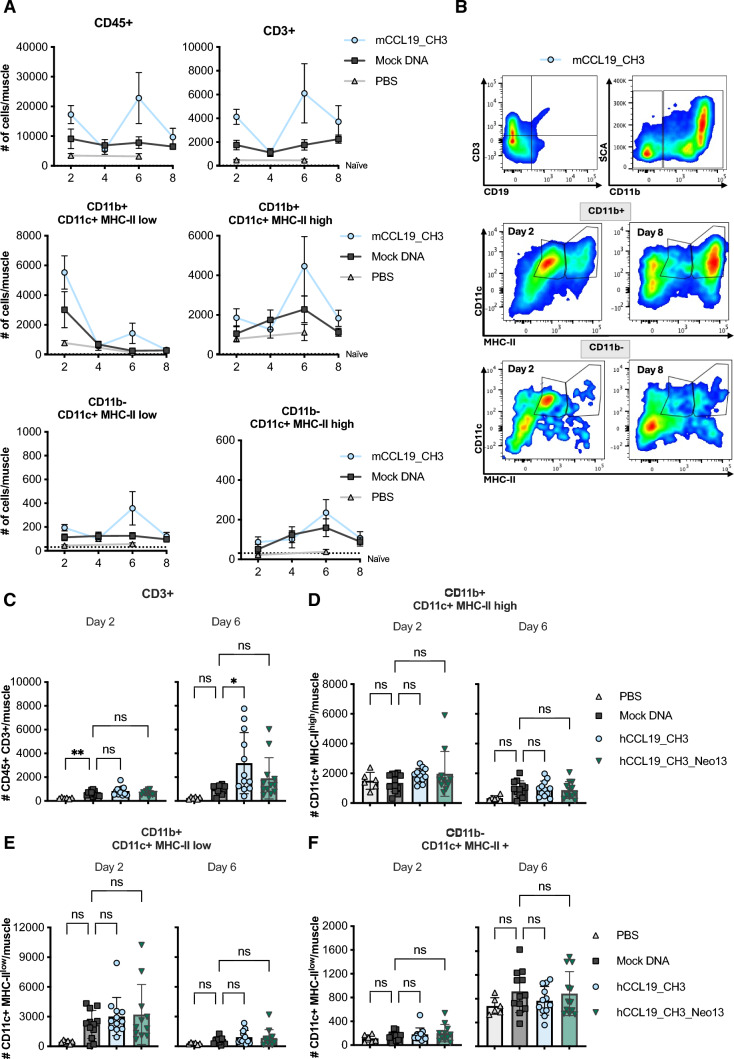
Recruitment of immune cells to the immunization site by mCCL19_CH3-derived DNA constructs **A** Quantification of immune cells per muscle at two, four, six, or eight days after one i.m. immunization with 90 μg of DNA formulated with poloxamer 188. The number of immune cells (CD45+), T-cells (CD45+CD3+CD19-), and DCs (CD45+CD3-CD19-CD11b+CD11c+MHC-II+) were quantified by flow cytometry (mean ± SD, 34 mice distributed as 2–3 mice/group, n (muscle lysates) = 4–6). **B** Gating strategy for A. **C**–**E** Quantification of immune cells per muscle two and six after one i.m. immunization with 90 μg of DNA in a subsequent experiment(mean ± SD, 42 mice distributed as 3–6 mice/group, n (musclelysates) = 6–12). Statistics (**C**–**E**): One-way ANOVA with Tukey’s correction for multiple tests. Significance: ns *p* > 0.05, *p < 0.05, **p < 0.01

The original article has been corrected.

