# Supplementary material for: APC-targeted DNA vaccines: the role of CCL19 in immune cell recruitment and early onset of the immune response
Source: Cancer Immunol Immunother. 2026 Mar 10;75(4):101. doi: 10.1007/s00262-026-04339-6 (PMC12976218; doi:10.1007/s00262-026-04339-6)
Supplement: Supplementary file 1 — Supplementary file1 (DOCX 575 kb) [file 262_2026_4339_MOESM1_ESM.docx]

# APC-targeted DNA vaccines: The role of CCL19 in immune cell recruitment and early onset of the immune response

## Supplementary material

**Supplementary Figure 1. Immunogenicity and anti-tumor efficacy of cancer neoantigens improve upon adding a secretion signal and CCL19 as an APC-targeting unit** (**A**) Schematic study timeline. BALB/c mice (n =13 mice per group) received five weekly immunizations of 3 or 6 μg of DNA prophylactically. Two weeks after the first immunization, mice were inoculated with 2 x 10^5^ CT26 tumor cells s.c. in the right flank (defined as study day 0). On study day 4, the frequency of C1-specific T cells was evaluated in peripheral blood. (**B**) Mean tumor volume (mm3) +/- SEM with last observation carried forward (LOCF) over time and mean AUC +/- SD of individual tumors for each treatment group. (**C**) Frequency of C1-specific CD8+ T cells. Mean +/- SD (n=6-7 mice per group). Statistics: Kruskal-Wallis test and Dunn ́s multiple comparison test. All comparisons performed are displayed. *p< 0.0, ****p< 0.0001.

**Supplementary Figure 2. Flow cytometry analysis of peripheral blood and a gating strategy for identifying cytokine-secreting T cells after ICS.** Single cells were selected based on their forward scatter (FSC) and side scatter (SSC) properties. Then, dead cells were excluded using a live/dead dye. Live single cells were then gated on CD3+ expression to identify T cells and further analyzed based on expression of CD4+ and CD8+. The percentage of reactive T cells in each subset was determined using the cytokine markers IFN-γ and TNF-α.

**Supplementary Figure 3. Flow cytometry analysis of peripheral blood and gating strategy for identifying antigen-specific CD8+ T cells.** After selecting single cells based on their forward scatter (FSC) and side scatter (SSC) properties, CD8+ T cells are selected based on the co-expression of CD3 and CD8 markers. The frequency of vaccine-specific T cells is analyzed as the percentage of C1-specific CD8+ T cells using a fluorochrome-labeled MHC-I C1-specific tetramer.
